# Supplementary material for: In situ redox reactions facilitate the assembly of a mixed-valence metal-organic nanocapsule
Source: Nat Commun. 2018 May 29;9:2119. doi: 10.1038/s41467-018-04541-w (PMC5974140; doi:10.1038/s41467-018-04541-w)
Supplement: Supplementary file 1 — Supplementary Information [file 41467_2018_4541_MOESM1_ESM.pdf]

Supplementary Information

In-situ Redox Reactions Facilitate the Assembly of a Mixed-Valence Metal-Organic Nanocapsule

Rathnayake *et al.*

Supplementary Methods:

Synthesis of *C*-pentylpyrogallol[4]arene (PgC_5)

Pyrogallol (25 g. 0.21 mol) was dissolved in 95% ethanol (40 ml) in a 250 ml round-bottomed flask. Hexanal (25 ml, 0.20 mol) and concentrated HCl (1 ml) was added to the mixture and refluxed overnight under nitrogen. The resultant white crystalline product was filtered and dried under vacuum. The final product was used for the synthesis without further purification.

Synthesis of 1

PgC_5 (0.166 g, 0.2 mmol) and sodium methoxide (0.032 g, 0.6 mmol) were mixed in a 1:1 (v/v) CH_2Cl_2/ethanol mixture (10 ml each). The solution was sonicated at 45°C for 20 minutes. The resulted pinkish-white turbid solution was cooled down for 10-15 minutes and $Mn(NO_3)_2$. 4 H_2O (0.2002 g, 0.8 mmol) was added. The solution was sonicated for an additional 30 minutes at 45°C (Final pH = 3.84). The resulting dark solution was set aside and black crystals formed over a period of two days upon slow evaporation of the mother liquor. Yield: 0.86 g = 6 % (with respect to Mn).

Crystal data for 1: $C_{288}H_{312}Mn_{25}N_6O_{120}$, M = 7150.92, Black prism 0.400 x 0.400 x 0.300 mm, Trigonal space group = $R3c$, a = 42.0083(1), c = 43.0332(2) Å, α = 90°, β = 90°, γ = 120°, V = 65766(5) $Å^3$, Z = 6, D_c = 1.083 g/cm^3, F_{000} = 22002, Synchrotron λ = 0.7749 Å, T = 100(2)K, $2\theta_{max}$ = 57.3º, 288601 reflections collected, 28928 unique (R_{int} = 0.0371) Final GOF = 1.084, $R1$ = 0.0789, $wR2$ = 0.2231 R indices based on 23164 reflections with I > 2σ(I) (refinement on F^2), 1145 parameters, 73 restraints. Lp and absorption corrections applied (μ = 0.954 mm^{-1}).

Supplementary Figures:

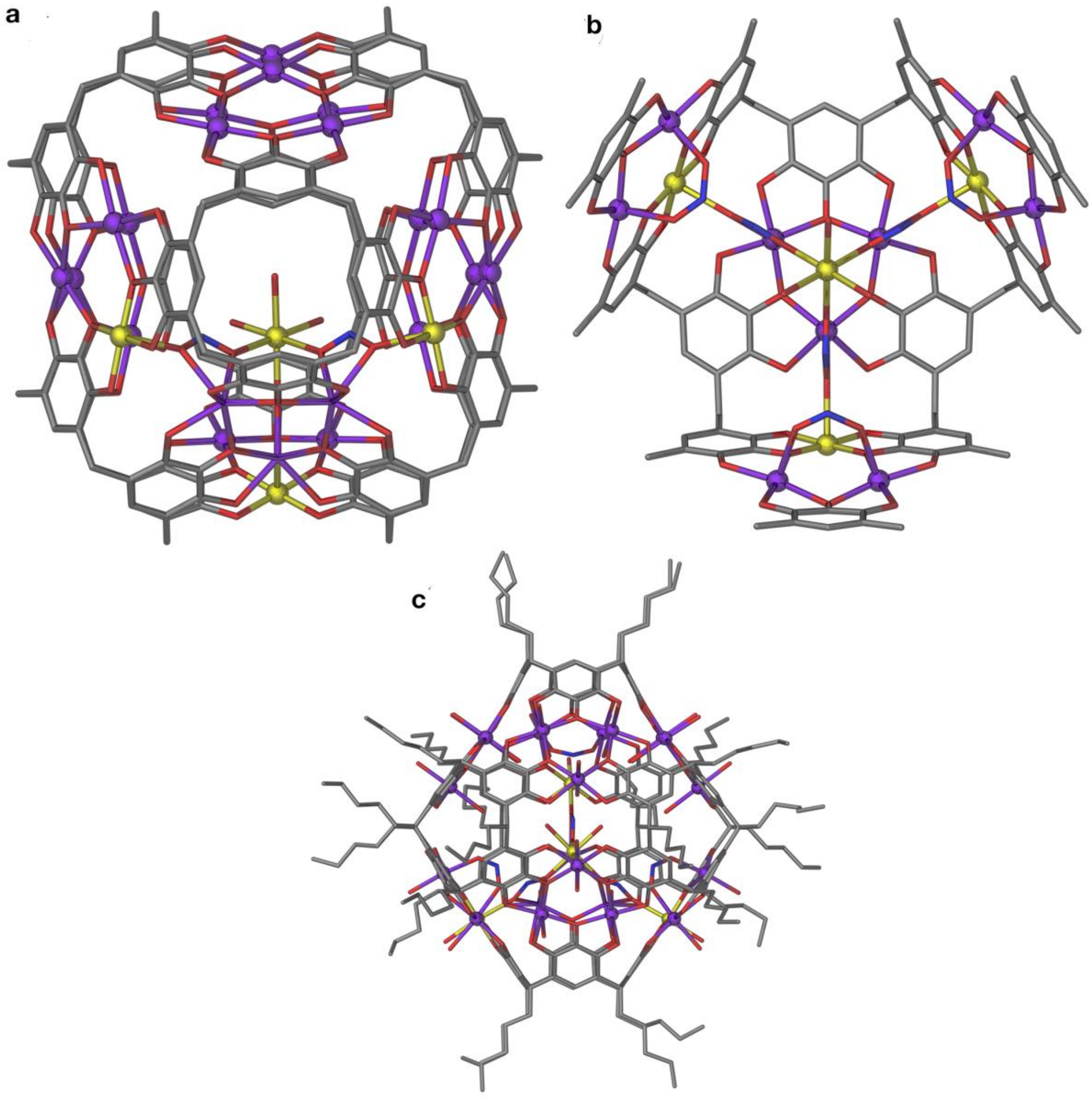

Supplementary Figure 1: **Different views of 1 in the solid-state. a** An alternative side view of **1** showing the distribution of Mn^{II} and Mn^{III} in the MONC. **b** Top-down view of a part of **1** showing the coordination of central Mn^{III} to framework Mn^{III} ions on adjacent facets via μ-NO_2^- bridges. **c** A side view of **1** showing the disordered pentyl groups. Color code: Mn^{II} – purple, Mn^{III} – yellow, N – blue, O – red, C – grey.

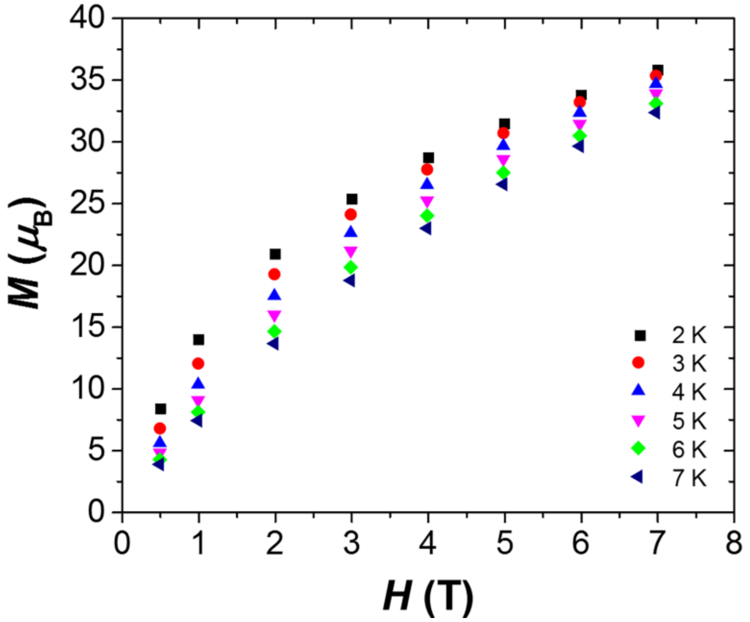

Supplementary Figure 2: **Magnetization analysis for compound 1.** Magnetization (*M*) vs. field (*H*) for **1** in the indicated field and temperature ranges.

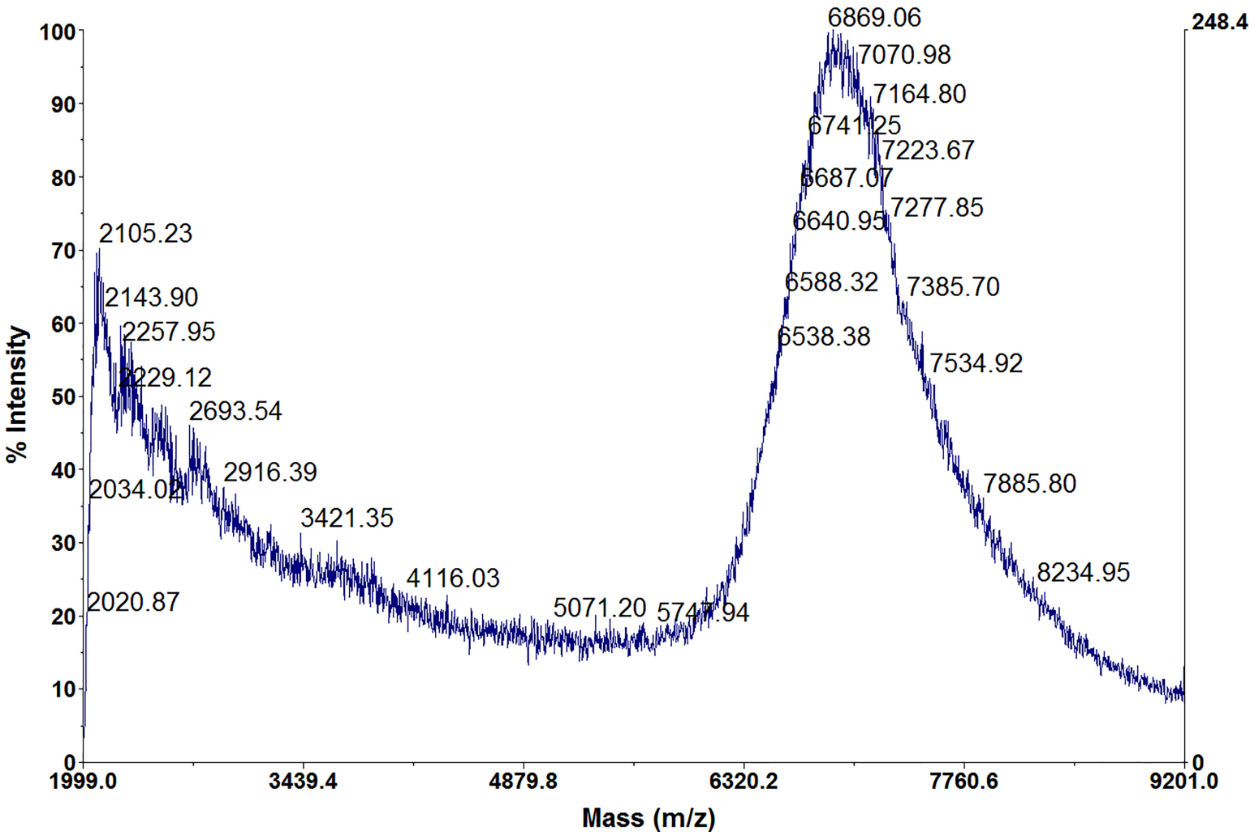

Supplementary Figure 3: **MALDI-TOF mass spectrometry analysis of 1.** The spectrum was obtained using dithranol as the matrix. The intense peak at 6869.06 Da represents the ionic fragment of $[Mn_{25}(PgC_5)_6(NO_2^-)_6(H_2O)_9\ (C_2H_5OH)_3]^+$ ($C_{294}H_{348}O_{96}N_6Mn_{25}$, calculated m/z = 6869.45 Da). The peak at 2105.23 Da is likely be due to an ionic fragment of two adjacent facets at the concave site $[Mn_7(C_{72}H_{78}O_{26}N_4)(CH_2Cl_2)_2(C_2H_5OH)_3]^+$ ($C_{80}H_{100}O_{29}Cl_4N_4Mn_7$, calculated m/z = 2106.36 Da) (Fig. 2b). Some other peaks have also been assigned as listed in Supplementary Table 2.

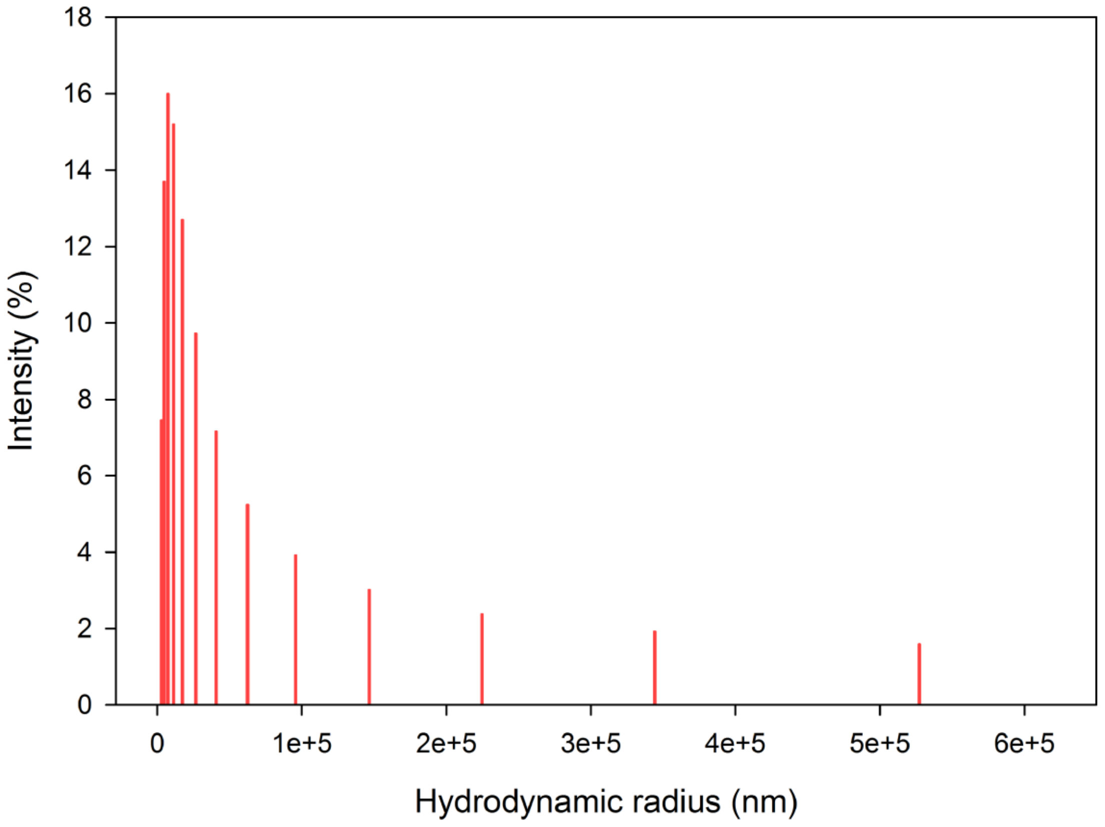

Supplementary Figure 4: **Dynamic light scattering (DLS) analysis of 1 in CH_2Cl_2.** The DLS analysis of **1** shows a monomodal polydisperse behavior. This observation indicates that, in CH_2Cl_2, **1** prefers to exist as large aggregates, with different hydrodynamic radii, rather than individual species.

Supplementary Tables:

	BVS value	Assignment
**Mn1**	2.32	Mn^{II}
**Mn2**	2.39	Mn^{II}
**Mn3**	2.43	Mn^{II}
**Mn4**	2.14	Mn^{II}
**Mn5**	2.18	Mn^{II}
**Mn6**	3.31	Mn^{III}
**Mn7**	2.03	Mn^{II}
**Mn8**	2.18	Mn^{II}

Supplementary Table 1: **Bond valence sum analysis for Mn1-Mn8 ions and the corresponding oxidation state assignments.**[1]

Found m/z (Da)	Calculated m/z (Da)	[Fragment]$^+$	Formula
7534.92	7534.45	$[Mn_{25}(PgC_5)_6(NO_2^-)_6 (H_2O)_{30} (CH_2Cl_2)_5]^+$	$C_{293}H_{382}O_{114}Cl_{10}N_6Mn_{25}$
7385.70	7286.35	$[Mn_{25}(PgC_5)_6(NO_2^-)_6 (H_2O)_{30} (CH_2Cl_2)(C_2H_5OH)_2]^+$	$C_{293}H_{386}O_{116}Cl_2N_6Mn_{25}$
7277.85	7276.35	$[Mn_{25}(PgC_5)_6(NO_2^-)_6 (H_2O)_{32} (CH_2Cl_2)(C_2H_5OH)]^+$	$C_{291}H_{384}O_{117}Cl_2N_6Mn_{25}$
7223.67	7223.16	$[Mn_{25}(PgC_5)_6(NO_2^-)_6 (H_2O)_8 (CH_2Cl_2)_6]^+$	$C_{294}H_{340}O_{92}Cl_{l2}N_6Mn_{25}$
7164.80	7163.47	$[Mn_{25}(PgC_5)_6(NO_2^-)_6 (H_2O)_{21} (CH_2Cl_2)_2(C_2H_5OH)]^+$	$C_{292}H_{364}O_{106}Cl_4N_6Mn_{25}$
7070.98	7070.53	$[Mn_{25}(PgC_5)_6(NO_2^-)_6 (H_2O)_{18} (CH_2Cl_2)(C_2H_5OH)_2]^+$	$C_{293}H_{362}O_{104}Cl_2N_6Mn_{25}$
6741.25	6741.45	$[Mn_{25}(PgC_5)_6(NO_2^-)_6 (H_2O)_7 (C_2H_5OH)]^+$	$C_{290}H_{332}O_{92}N_6Mn_{25}$
6687.07	6687.45	$[Mn_{25}(PgC_5)_6(NO_2^-)_6 (H_2O)_4 (C_2H_5OH)]^+$	$C_{294}H_{326}O_{89}N_6Mn_{25}$
6640.95	6641.45	$[Mn_{25}(PgC_5)_6(NO_2^-)_6 (H_2O)_4]^+$	$C_{288}H_{320}O_{88}N_6Mn_{25}$

Supplementary Table 2: **Structural assignment of peaks in the MALDI-TOF spectrum.**

Supplementary References:

[1] I. Brown, D. Altermatt, *Acta Cryst. B* **1985**, *41*, 244-247.
